# Potency of Bone Marrow-Derived Mesenchymal Stem Cells and Indomethacin in Complete Freund's Adjuvant-Induced Arthritic Rats: Roles of TNF-*α*, IL-10, iNOS, MMP-9, and TGF-*β*1

**DOI:** 10.1155/2021/6665601

**Published:** 2021-04-05

**Authors:** Eman A. Ahmed, Osama M. Ahmed, Hanaa I. Fahim, Tarek M. Ali, Basem H. Elesawy, Mohamed B. Ashour

**Affiliations:** ^1^Physiology Division, Zoology Department, Faculty of Science, Beni-Suef University, P.O. Box 62521, Beni-Suef, Egypt; ^2^Department of Physiology, College of Medicine, Taif University, P.O. Box 11099, Taif 21944, Saudi Arabia; ^3^Department of Physiology, Faculty of Medicine, Beni-Suef University, Beni-Suef, Egypt; ^4^Department of Clinical Laboratory Sciences, College of Applied Medical Sciences, Taif University, P.O. Box 11099, Taif 21944, Saudi Arabia; ^5^Department of Pathology, Faculty of Medicine, Mansoura University, Egypt

## Abstract

Rheumatoid arthritis (RA) is an autoimmune syndrome affecting joint spaces, leading to the disabled state. Currently, there is no optimal therapy for RA except for systemic immunosuppressants that have variable undesirable effects after long-term use. Hence, the need for other treatment modalities has emerged in an attempt to develop a treating agent that is effective but without bad effects. Bone marrow-derived mesenchymal stem cells (BM-MSCs) may be an alternative medicine since they may differentiate into a variety of mesenchymal tissues including bone and cartilage. Indomethacin (IMC) could be suggested as an analgesic, anti-inflammatory, and antirheumatic potential agent against the course of RA since it possesses significant palliative effects and antipyretic properties. Therefore, our target of this study was to explore and compare the effect of BM-MSCs (1 × 10^6^ cells/rat at the 1^st^, 6^th^, 12^th^, and 18^th^ days) and IMC (2 mg/kg b.w./day for 3 weeks) either alone or in combination on arthritic rats. The model of rheumatoid arthritis in rats was induced by subcutaneous injection of 0.1 mL/rat CFA into the footpad of the right hind paw. The BM-MSC intravenous injection and IMC oral administration significantly reduced the elevated right hind leg paw diameter and circumference, serum anti-CCP, and ankle joint articular tissue expressions of TNF-*α*, iNOS, MMP-9, and TGF-*β*1 while they significantly increased the lowered articular IL-10 expression in CFA-induced arthritic rats. The combinatory effect of the two treatments was the most potent. In conclusion, the treatment of RA with BM-MSCs and IMC together is more effective than the treatment with either BM-MSCs or IMC. The Th1 cytokine (TNF-*α*), Th2 cytokine (IL-10), iNOS, MMP-9, and TGF-*β*1 are important targets for mediating the antiarthritic effects of BM-MSCs and IMC in CFA-induced arthritis in rats.

## 1. Introduction

Rheumatoid arthritis (RA) is a syndrome of ongoing inflammation that is categorized with joint rubefaction, edema, and impairment of synovial joints. Such phase is correlated with inflammatory cell proliferation and penetration of the synovium, in addition to bone as well as pariarticular cartilage dysfunction [1]. RA is considered a chief cause of permanent disability, augmented mortality, and socioeconomic costs [2]. Its prevalence is around 1% of the global population and is in continuous increase with time [3] and propagates in females 3 times more than males which could be attributed to sex hormones. It is also linked with the extra-articular manifestations involving renal, pulmonary, and cardiovascular problems [4]. Former research and studies suggested that the imbalanced immunological responses in addition to genetic factors play a fundamental role in RA development. The mechanism of RA pathogenesis and its etiology remains generally indefinite. However, it primarily is activated by T cell immunological responses that release various proinflammatory mediators [5] such as tumor necrosis factor-alpha (TNF-*α*), matrix metalloproteinase-9 (MMP-9), inducible nitric oxide synthase (iNOS), and transforming growth factor-beta-1 (TGF-*β*1). Also, the anticyclic citrullinated protein antibodies (anti-CCP) are subsequently produced inducing local edema, inflammation, and ultimately joint destruction [6]. In comparison, a compensatory anti-inflammatory response in the RA synovia is also evidenced by producing anti-inflammatory cytokines such as IL-10 that is believed to suppress RA progression [7]. Accordingly, it became so critical to explore promising mechanisms and seek potential safer alternative therapies to improve the inflammatory pathological progress in RA patients [8].

There are many common drugs administered for pain relief and delay of RA progression including traditional nonsteroidal anti-inflammatory drugs (NSAIDs) combined with those steroids or disease-modifying antirheumatic drugs (DMARDs), also hormonal-based drugs or corticosteroids, and the novel biological therapeutic agents, such as the tumor necrosis factor-*α* (TNF-*α*) antibody and the decoy TNF-*α* receptor [9]. However, the application of these available medicines is frequently limited and undesired by patients due to their high costs, and their administration for a long time is accompanied by the incidence of harm and extensive side effects [10]. In this regard, unconventional therapies or anti-inflammatory substances from other different sources that provide an effective but safer treatment of arthritis have aroused great public interest in recent years [11]. Various experimental animal models have been well known in rats to study the disease initiation and propagation as well as determine the probable efficacy of antiarthritic and anti-inflammatory agents [12]. The arthritis model induced *via* complete Freund's adjuvant (CFA) reagent is one of the best available models for chronic inflammation and polyarthritis with features that resemble human RA and is still widely used in the preclinical testing of arthritis [13–15]. Mesenchymal stem cells (MSCs) are multipotent cells that differentiate into various kinds of cells including adipocytes, osteoclasts, and chondrocytes. They could be extracted from numerous mesodermal tissues such as the dental pulp, placenta, umbilical cord blood, menstrual fluid, umbilical cord, adipose tissue, and bone marrow [16]. They were found to exert immunosuppressive purposes on both the innate and adaptive immune cells [17]. Consequently, MSCs have an interesting therapeutic cell candidate for tissue engineering and repair of damaged structures in autoimmune diseases such as RA. This could be attributed to their anti-inflammatory and regenerative functions besides their capacity to attenuate the exacerbated pathogenic immune response observed in these patients [17].

Moreover, indomethacin (IMC), 1-(p-chlorobenzoyl)-5-methoxy-2-methylindole-3-acetic acid, is considered a nonsteroidal indole derivative with anti-inflammatory activity and chemopreventive properties. As a nonsteroidal anti-inflammatory drug (NSAID), indomethacin reduces prostaglandins by inhibiting cyclooxygenase (COX) enzymes, COX-1 and COX-2, with greater selectivity for COX-1. IMC inhibits COX enzymes by binding to them, forming COX-IMC complexes [18, 19]. Also, IMC exhibits potent antipyretic effects and analgesic properties that may enable it to relieve the pain of patients and overcome the inflammatory reactions of the disease. The Food and Drug Administration (FDA) approved its use for many diseases including primary dysmenorrhea, pericarditis, juvenile arthritis, pseudogout, and Paget's disease [20]. It has acquired an established place in the treatment of osteoarthrosis of the hip. It was introduced in 1963 for the treatment of ankylosing spondylitis and seems to be effective in degenerative joint diseases. Also, it showed benefit in treating acute gout and musculoskeletal disorders, inflammation, and edema [21]. Additionally, IMC has been used by clinicians in treating RA and preventing its progression. However, it is rarely used solely but usually showed greater efficacy in conjunction with DMARDs such as adalimumab, etanercept, infliximab, and methotrexate [20].

In conductance with the previous publications, this study was designed to evaluate the convenience and bioavailability of BM-MSCs and IMC administered in combination to associate the advantages of both of them in relation to each treatment (BM-MSCs or IMC) alone, *via* their role in suppressing the Th1 (TNF-*α*, iNOS, MMP-9, and TGF-*β*1) pathway while promoting the Th2 (IL-10) pathway and subsequently overcoming the course of the disease in the CFA-arthritic rat model.

## 2. Materials and Methods

### 2.1. Animal Procurement and Maintenance

Our experiment included 50 male Wistar rats (120-150 g, weight; 10-12 weeks, specific pathogen-free) that were obtained from VACSERA (Helwan Station, Cairo, Egypt). The animals were kept in an animal facility at temperature 22 ± 2°C, relative humidity 55 ± 5%, and 12-hour (h)/12 h light/dark cycle. The animal experiment was approved by the local committee for animal experimentation, Faculty of Science, Beni-Suef University, Egypt (ethical approval number: BSU/FS/2017/11).

#### 2.1.1. Induction of Arthritis

For arthritis induction, animals were inoculated by subcutaneous injection of 0.1 mL/rat CFA solution (Sigma Chemical Co., St. Louis, MO, USA) into the footpad of the right hind paw as described by Ahmed et al. [13] for two consecutive days. Each 1 mL of CFA contains 1 mg of *Mycobacterium tuberculosis*, heat-killed and dried, 0.85 mL paraffin oil, and 0.15 mL mannide monooleate.

#### 2.1.2. Animal Grouping

The experimental model was designed as described in our recent study [22] as follows:

*Group 1 (normal)*. It consists of healthy rats that were given the equivalent volumes of carboxymethylcellulose (CMC) daily and orally for 3 weeks and Dulbecco's modified Eagle's medium (DMEM) intravenously at the 1^st^, 6^th^, 12^th^, and 18^th^ days.

*Group 2 (CFA)*. It is composed of CFA-induced arthritic rats and was orally given the equivalent volumes of CMC daily and orally for 3 weeks and DMEM intravenously at the 1^st^, 6^th^, 12^th^, and 18^th^ days.

*Group 3 (CFA+BM-MSCs)*. This group consists of CFA-induced arthritic rats that received four doses of BM-MSCs (1 × 10^6^ cells/rat/dose) by intravenous injection through the lateral tail vein per rat [23]. Each dose was suspended in 0.2 mL DMEM (Dulbecco's modified Eagle's medium). Doses were given on the 1^st^, 6^th^, 12^th^, and 18^th^ days after CFA injection.

*Group 4 (CFA+IMC)*. This group is composed of CFA-induced arthritic rats supplemented orally with IMC in a dose of 2 mg/kg body weight (b.w.)/day for 3 weeks after CFA injection. IMC was freshly prepared immediately before administration by dissolving in 5 mL of 1% CMC for three weeks. IMC was acquired from Sigma Chemical Company (Sigma Chemical Co., St. Louis, MO, USA).

*Group 5 (CFA+BM-MSCs+IMC)*. This group consists of CFA-induced arthritic rats that were concurrently supplemented with BM-MSCs and IMC as described in groups 3 and 4.

### 2.2. Isolation and Culture of BM-MSCs

The isolating and culturing technique of the BM-MSCs is established on the approach of Chaudhary and Rath [24] and our former publications [22, 25].

### 2.3. Evaluation of Paw Edema and Swelling Rate in Arthritis

In the present study, for evaluating the arthritis development, the paw circumference (cm) and the paw diameter (mm) of the right hind paw were used as indicators of the rate of swelling and joint edema. Measurements were obtained at various times on days 0, 7, 14, and 21 after CFA induction. The joint diameter was recorded with a microtome screw gauge [26], while the paw circumference was evaluated by wrapping a string around the paw and then measuring its length on a ruler. Edema and the swelling rate for the CFA rats were compared to those for a normal control group, while those for the treated rats were compared to those for the CFA group. The rats were anesthetized by ether inhalation before measurement.

### 2.4. Measurement of Anti-CCP and IL-10 Using the ELISA Technique

Serum anti-CCP and IL-10 levels were determined in different groups using specific enzyme-linked immunosorbent assay (ELISA) kits purchased from R&D Systems (R&D Systems, Inc., Minneapolis, MN, USA) according to the manufacturer's instructions.

### 2.5. Determination of the Expression of Various Genes by RT-PCR

The mRNA expression levels of TNF-*α*, MMP-9, and iNOS in relation to the housekeeping gene beta-actin (*β*-actin) were determined using reverse transcription polymerase chain reaction (RT-PCR).

#### 2.5.1. Ribonucleic Acid (RNA) Isolation

The RNA product was extracted totally from ankle joints using the Thermo Scientific GeneJET RNA extraction kit purchased from Thermo Fisher Scientific Inc., Rochester, New York, USA [27]. In liquid nitrogen, samples were homogenized and then lysed using a lysis buffer solution that consists of guanidine thiocyanate and a chaotropic salt which protects RNA from endogenous RNases. The lysate was then mixed with ethyl alcohol and mounted on a purification column. Both the chaotropic salt and the ethyl alcohol made RNA bind to the silica membrane as the lysate is spun through the column. Impurities were subsequently removed away from the membrane by washing the column with a washing buffer solution. Then, pure RNA was eluted with a nuclease-free water reagent in low-ionic strength conditions. And the amount of purified RNA was quantified by using a UV spectrophotometer according to the following formula: RNA *μ*g/*μ*L = O.D.260 nm × (40 *μ*g RNA/mL) × dilution factor/1000. To ensure the high purity of the isolated RNA, we checked the purity of RNA that ranged between 1.8 and 2.0. By the end, 0.5 *μ*g of purified RNA was used for the production of complementary deoxyribonucleic acid (cDNA) that was kept at -20°C, for further assay of the mRNA.

#### 2.5.2. Reverse Transcription Polymerase Chain Reaction (RT-PCR) Analysis

RT-PCR analysis was performed as described in Ahmed et al.'s [22] research work, and the relative expression level of TNF-*α*, iNOS, and MMP-9 was normalized to the *β*-actin housekeeping gene. All the primers used in this experiment were synthesized by Sangon Biotech (Shanghai, China) (Table 1).

### 2.6. Western Blot Analysis

The amount of TGF-*β*1 protein was assayed using the Western blot technique. Briefly, we used the ice-cold RIPA lysis buffer to extract the proteins from joint tissue. The Bradford Protein Assay Kit (SK3041) for quantitative protein analysis was provided by Bio Basic Inc. (Markham, Ontario, L3R 8T4, Canada). A Bradford assay was performed according to the manufacturer's instructions to determine protein concentration in each sample. Equivalent amounts (30 *μ*g) of protein were divided using 10% sodium dodecyl sulfate-polyacrylamide gel electrophoresis (SDS-PAGE). Next, the proteins loaded on the gel were shifted onto membranes of polyvinylidene fluoride (PVDF). Then, overnight, the membrane was probed at 4°C with the TGF-*β*1-specific primary antibody (cat. no. 9574; Thermo Fisher Scientific). After washing with Tris-buffered saline with Tween 20 (TBST) three times, the blots were prepared for incubation with horseradish peroxidase-conjugated secondary antibodies (1 : 5000, Santa Cruz Biotechnology, CA) at RT 25°C for 30 minutes. The blots were washed again, and then, the signal of the chemiluminescence was visualized with an X-ray film [22, 28].

### 2.7. Histopathological Examination

On day 21 of arthritis induction and after euthanization, the right hind leg ankle joints of 4 rats from each group were detached and conserved for 48 hours in 10% buffered formalin. Decalcification of the sample tissues was performed using paraffin blocks with 10% nitric acid for 2 weeks. Finally, 5 *μ*m thick cross sections of these blocks were dyed with hematoxylin-eosin and viewed using a light microscope to determine the histopathological changes and severity of arthritis.

### 2.8. Statistical Analysis

Statistical tests were performed utilizing IBM SPSS Statistics program version 22.0 (IBM, Armonk, NY, USA). All values were represented as the mean and standard error of the mean (mean ± SE). Differences among groups were estimated for statistical significance using the one-way analysis of variance (ANOVA) test followed by the Tukey–Kramer post hoc test for comparisons between groups, and *p* < 0.05 was considered the minimal level of significance [29].

## 3. Results

### 3.1. Effect of Treatments on Paw Edema

**Figure 1:**
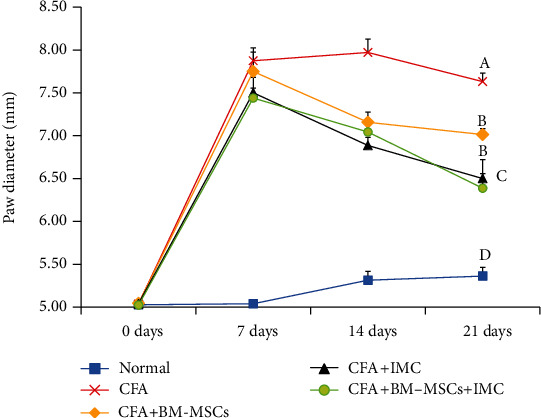
Effect of BM-MSCs and/or IMC on the right hind paw diameter (mm) in CFA-induced rats. Means, which have different symbols, A, B, C, and D, are significantly different at *p* < 0.05.

**Figure 2:**
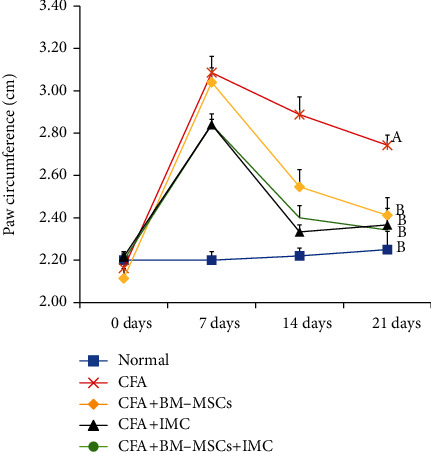
Effect of BM-MSCs and/or IMC on the right hind paw circumference (cm) in CFA-induced rats. Means, which have different symbols, A and B, are significantly different at *p* < 0.05.

All rats developed arthritis after adjuvant injection. The CFA-induced arthritic rats showed a statistically significant (*p* < 0.05) increase in the paw diameter and circumference (edema) that was maintained for 21 days compared with a normal control group (Figures 1 and 2). However, the arthritic treated rats administered with BM-MSCs and/or IMC showed a significant (*p* < 0.05) decrease in those parameters by the end of the experiment with inhibition percentages of 8.10, 14.83, and 16.30% and 12.07, 14.60, and 13.72% for the paw diameter and circumference, respectively, in comparison with CFA rats.

### 3.2. Effect of Treatments on Anti-CCP and IL-10 Concentrations

**Figure 3:**
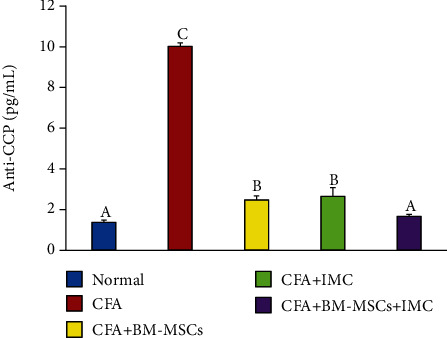
Effect of BM-MSCs and/or IMC on anti-CCP concentration in CFA-induced rats. Means, which have different symbols, A, B, and C, are significantly different at *p* < 0.05.

**Figure 4:**
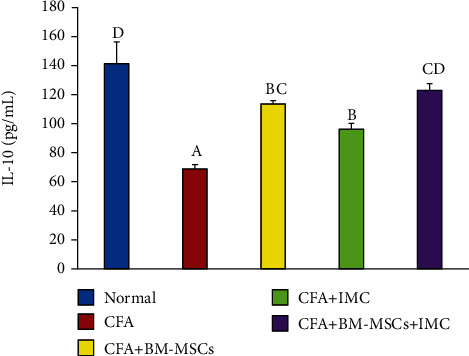
Effect of BM-MSCs and/or IMC on IL-10 concentration in CFA-induced rats. Means, which have different symbols, A, B, C, and D, are significantly different at *p* < 0.05.

Levels of anti-CCP and IL-10 were detected in serum using a standard ELISA technique (Figures 3 and 4), respectively. Rats immunized with CFA exhibited a significant (*p* < 0.05) increase in the anti-CCP autoantibody (631.71%) but a marked reduction in anti-inflammatory IL-10 cytokine levels (-51.29) compared with the normal control group. Conversely, administration of BM-MSCs, IMC, and BM-MSCs+IMC each, respectively, successfully decreased the anti-CCP level (-75.33, -73.50, and -83.33) and promoted IL-10 production (64.95, 39.55, and 78.43%) as well when compared to the normal group.

### 3.3. Evaluation of TNF-*α*, iNOS, and MMP-9 mRNA Expression Level and Protein Level of TGF-*β*1 in Ankle Joint Articular Tissues

**Figure 5:**
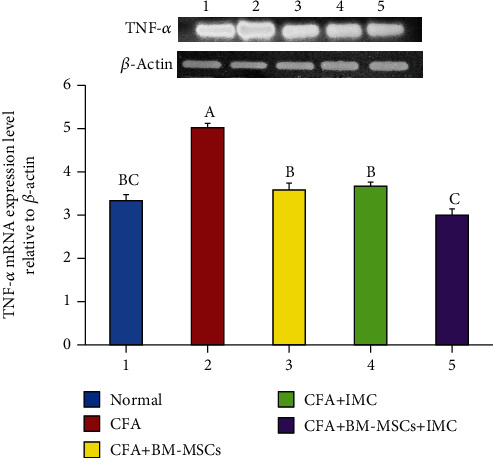
Effect of BM-MSCs and/or IMC on the TNF-*α* expression level relative to *β*-actin in CFA-induced rats. Means, which have different symbols, A, B, and C, are significantly different at *p* < 0.05.

**Figure 6:**
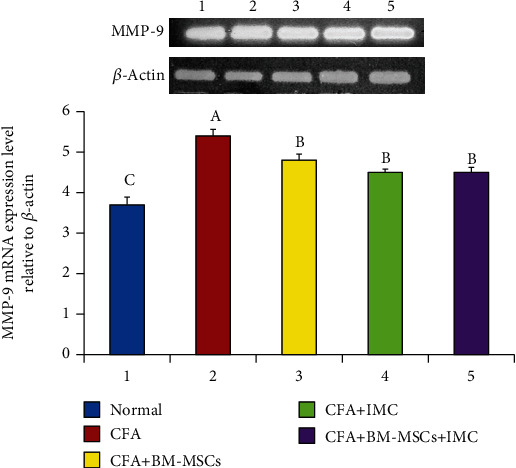
Effect of BM-MSCs and/or IMC on the MMP-9 expression level relative to *β*-actin in CFA-induced rats. Means, which have different symbols, A, B, and C, are significantly different at *p* < 0.05.

**Figure 7:**
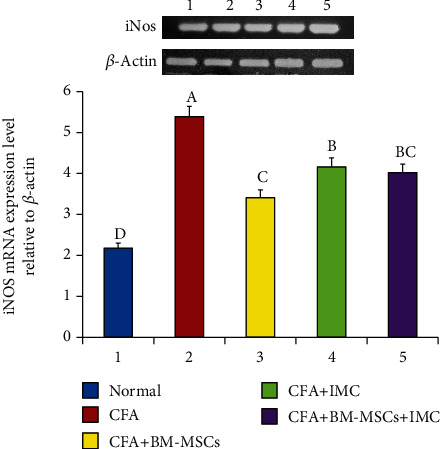
Effect of BM-MSCs and/or IMC on the iNOS level mRNA expression relative to *β*-actin in CFA-induced rats. Means, which have different symbols, A, B, C, and D, are significantly different at *p* < 0.05.

**Figure 8:**
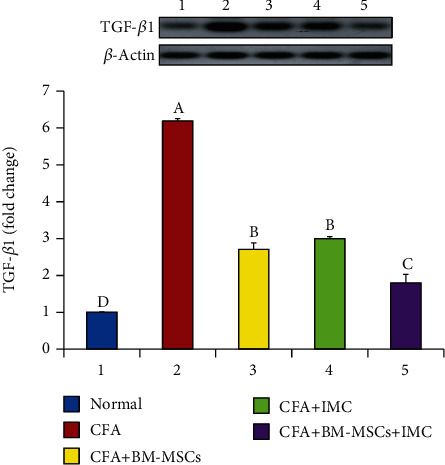
Effect of BM-MSCs and/or IMC on the TGF-*β*1 level in CFA-induced rats. Means, which have different symbols, A, B, C, and D, are significantly different at *p* < 0.05.

As represented in Figures 5–7, the TNF-*α*, MMP-9, and iNOS mRNA expression levels, respectively, in ankle joint articular tissues, were determined by the PCR technique. The arthritic untreated rats noticeably showed upregulation of their mRNA expression levels as compared to the normal ones. On the other hand, the rats treated with BM-MSCs and/or IMC showed apparent downregulation of their levels. Likewise, the TFG-*β*1 protein level was highly elevated in the arthritic group with a change percentage of 512.87% when compared with the normal control. However, the animals treated with BM-MSCs, IMC, and BM-MSCs+IMC, respectively, showed a significant reduction of its level with a change percentage of -56.22%, -51.53%, and -70.92%, respectively, concerning the arthritic control group (Figure 8).

### 3.4. Effect of Treatments on Gross Lesions (Macroscopic Changes) of the Right Hind Paw and Ankle Joint

**Figure 9:**
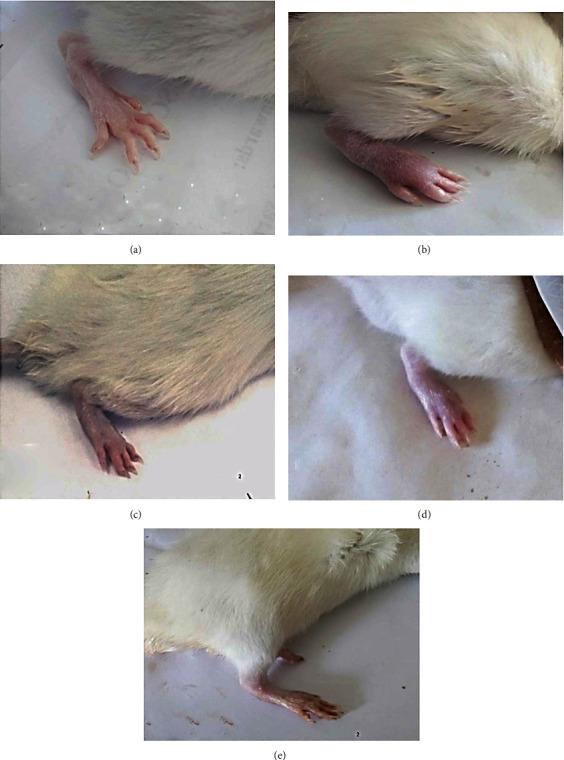
Effect of BM-MSCs and/or IMC on gross lesions on the right hind leg paw and ankle joint on day 21 post-CFA induction showing (a) normal rats, (b) CFA-induced arthritic control rats, (c) CFA+BM-MSC-treated rats, (d) CFA+IMC-treated rats, and (e) CFA+BM-MSC+IMC-treated rats.

Macroscopic changes such as edema and the swelling rate of the right hind paw and ankle joints acted as external features and inflammatory signs for evaluating the arthritic inflammatory model intensity. The CFA control group showed severe inflammation as well as paw and ankle joint swelling; on the other side, both of which gradually decreased following BM-MSC and/or IMC treatments by the end of the experiment (on day 21 post-CFA injection) (Figure 9).

### 3.5. Histopathological (Microscopic) Changes

**Figure 10:**
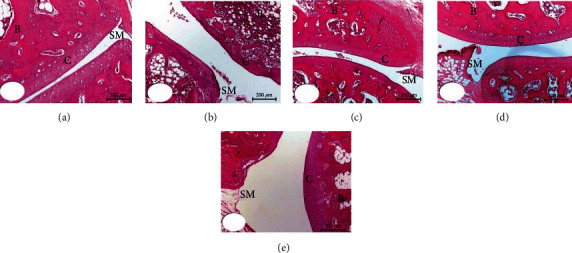
Photomicrographs (200x) of HE-stained sections showing the effect of BM-MSCs and/or IMC treatments on the histopathological changes in the ankle joints of CFA-induced arthritic rats. (a) Normal group. (b) CFA-arthritic control group. (c) CFA+BM-MSC-treated group. (d) CFA+IMC-treated group. (e) CFA+BM-MSC+IMC-treated group. SM: synovial membrane; C: cartilage; B: bone.

Histological sections of the right hind ankle joint obtained from normal rats showed a clear and complete histological architecture with the normal synovial membrane and normal articular (cartilage and bone) surfaces. The CFA-induced arthritic rats exhibited severe histological alterations including focal proliferation and degeneration of the synovial membrane forming the pannus that infiltrated with a massive number of mononuclear inflammatory cells, extensive and widespread erosion in the cartilage surface, and hypercellularity and hyperplasia of myeloid cells of the bone. On the contrary, sections of the CFA-induced arthritic rats treated with BM-MSCs and/or IMC presented highly improved histological configuration with nearly normal cartilage and bone surfaces except for slight inflammation of synovia that was moderate in IMC-treated rats and mild in both groups treated with BM-MSCs and those concurrently administered rats (BM-MSCs+IMC) (Figure 10).

## 4. Discussion

RA is regarded as a disabling autoimmune syndrome that is related to long-lasting joint inflammation besides extensive cartilage and bone impairment [30]. CFA is a widely used animal model for both researching pathogenesis and discovering novel therapies to treat RA in humans [31]. In the CFA-induced arthritis model, rats experience persistent swelling in several joints followed by inflammatory cell inflow, joint cartilage degradation, and bone integrity erosion and dysfunction. Herein, the diameter and the circumference of the right hind paw were estimated weekly and for 3 weeks as an index of the joint swelling, subsequently monitoring disease development besides the response to the tested drugs. In complete agreement with the study of Nagai et al. [32], our data displayed that paw edema and swelling reached the maximum on day 7 of arthritis induction in the acute phase (primary inflammation) and gradually declined until day 14 and then began the chronic phase of arthritis (secondary inflammation). By the end of the experiment (3^rd^ week), the arthritic control rats exhibited a significant increase in the paw diameter and circumference comparable to the normal rats. On the contrary, the BM-MSC- and/or IMC-treated rats efficiently inhibited the elevation about the arthritic control rats and were approximated to normal ranges. Consistent with our findings, the study of Porth [33] reported that edema of the right hind foot of adjuvant and arthritic rats immunized with low-dose IMC nanoparticle (0.4 mg/kg) oral administration was significantly lower regarding those immunized with a vehicle. Furthermore, these outcomes were strongly supported by the results of biochemical assays and revealed the anti-inflammatory efficacy of the tested drugs against CFA-induced arthritis.

Preceding research papers revealed that RA is initiated chiefly through immunological responses of T cells which induce cytokine release [33] and facilitate the development of autoantibodies, leading to joint destruction. Concerning the autoantibodies formed during the course of the disease, the anticitrullinated protein antibodies (ACPA) are the most common RA biomarker for diagnostics. It is produced as a response to the occurrence of autoantigens, named citrullinated peptides. These autoantigens could prompt local edema and inflammation *via* developing an immune response within the localized region of the joint [6]. Besides, the presence of ACPA is predictive for the development of a worse disease effect with more joint erosions along with time [34]. The recent investigation demonstrated that the sera of arthritic control rats showed a remarkable increase in the anti-CCP concentration level as compared to the normal group. Principally, the BM-MSC+IMC group besides BM-MSC- and IMC-supplemented rats clearly declined the elevated anti-CCP level compared to the arthritic control rats. Such an anti-CCP level proves the capabilities of the tested agents to modulate immune responses induced in RA, hence recommending them as promising antirheumatic drugs.

**Figure 11:**
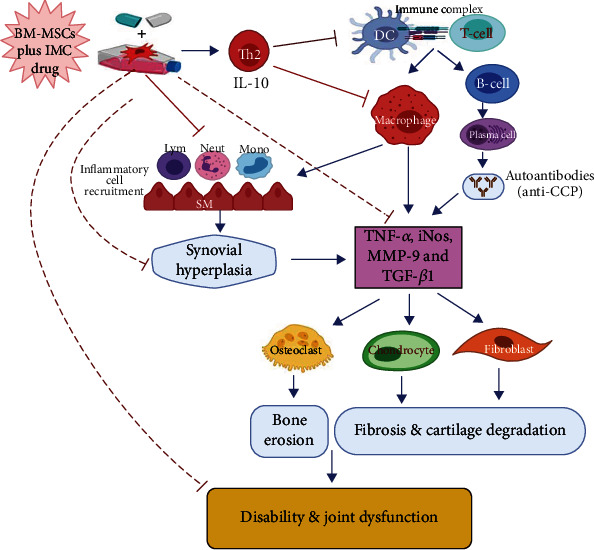
Schematic diagram showing the immunomodulatory effect of BM-MSCs+IMC administered concurrently in CFA-induced arthritis. IMC: indomethacin; BM-MSCs: bone marrow-derived mesenchymal stem cells; Th2: T helper 2; DC: dendritic cell; IL-10: interleukin 10; T cell: T lymphocytes; B cell: B lymphocytes; Mono: monocytes; Neut: neutrophils; SM: synovial membrane; iNOS: inducible nitric oxide synthase; TGF-*β*1: transforming growth factor-beta-1; TNF-*α*: tumor necrosis factor-alpha; anti-CCP: anticyclic citrullinated peptide antibody; MMP-9: matrix metalloproteinase-9.

Similarly, RA is considered an autoimmune disorder characterized by infiltration of immune cells (monocytes and lymphocytes). These inflammatory cells are deemed substantial in initiating and perpetuating RA as represented in Figure 11; it produces interleukins (ILs), as well as inflammatory mediators such as tumor necrosis factor-alpha (TNF-*α*), nitric oxide (NO), MMP-9, and prostaglandin E2 (PGE2) [35]. Those mediators are implicated in the inflammatory response and have various roles through many pathways. Therefore, modulating or blocking these pathways became the target of the new therapeutic tested drugs against the disease. In this study, we focused on TNF-*α*, iNOS, MMPs, and TGF-1*β* as originators of inflammation besides IL-10 as an inhibitor of inflammation within tissues.

Specifically, TNF-*α* is a principal cytokine that induces apoptosis in some cells and proliferative reactions in others and plays a crucial role in both acute and chronic inflammation [36]. It prompts the production of inducible nitric oxide synthase (iNOS) that in turn enhances the release of matrix metalloproteinases (MMPs). MMPs are a family of inflammatory mediators (MMP-3, MMP-13, and MMP-9) responsible for promoting extracellular matrix degradation and cartilage damage [37]. Therefore, when the TNF-*α* pathway is specifically blocked, the severity of inflammation is accordingly reduced; that is why it became a key therapeutic target to cease the evolution toward the chronic form of the disease [38]. In parallel, transforming growth factor-*β*1 (TGF-*β*1) is a component of the TGF-*β* superfamily of cytokines contributing to various cellular responses, such as apoptosis, proliferation, differentiation, and extracellular matrix production [39]. TGF-*β*1 is essential for the induction of RA-related fibrosis [40]. On the contrary, a compensatory anti-inflammatory response is also observed in RA synovial membranes. IL-10 is an upstream regulator and anti-inflammatory marker that is thought to control the progression of RA negatively. Several animal model studies of arthritis have illustrated the beneficial impact of IL-10 on reducing arthritis severity [7]. Similar to the explanation displayed in Figure 11 illustrating the IL-10 role in the course of the disease, Hisadome et al. [41] demonstrated that it controls the functioning of APCs and prevents cytokine release from activated macrophages. Also, van Roon et al. [42] established that IL-10 suppresses the production of protein lysing enzymes *via* monocytes that produced the inhibitor of metalloproteinase-1 (TIMP-1). Furthermore, it antagonized osteoclast formation (osteoclastogenesis) by suppressing the production of IL-6 in osteoclast precursors, hence overcoming the bone resorption induced by arthritis [43].

In addition to the former measurements, the anti-inflammatory impact of treatments on the CFA-induced arthritis model was more investigated macroscopically *via* evaluating the gross lesion changes and microscopically by demonstrating the histopathological changes on the right hind paw and ankle joint. Initially, histopathological or microscopic lesions of rats in the CFA control group exhibited an obvious synovial degradation and proliferation accompanied by cartilage erosion and bone mas resorption. Conversely, the BM-MSC-treated arthritic group and BM-MSC+IMC-treated arthritic group afforded significant protection against those alterations and exhibited a mild stage of inflammation while those supplemented by IMC displayed a moderate stage of inflammation. Correspondingly, the macroscopic lesions displayed intensive edema and paw swelling in the CFA-induced control rats that were interestingly improved in the BM-MSC+IMC-, BM-MSC-, and IMC-treated groups in respect to CFA.

Overall, in the current research, our data demonstrated a marked elevation of the proinflammatory TNF-*α* cytokine as well as the iNOS, MMP-9, and TGF-*β*1 gene expression levels in paw tissues of CFA-induced rats; however, the anti-inflammatory IL-10 levels in sera conversely declined as compared with the normal rats. As exhibited schematically in Figure 11, BM-MSC and IMC therapies either concurrently or alone received by the rats essentially downregulated the reported proinflammatory cytokines whereas promoted evidently the anti-inflammatory cytokine (IL-10) in comparison with the CFA-induced arthritic group. These results illustrated the ability of BM-MSCs and IMC to protect against cartilage and bone destruction, preventing further development of the disease through such immunoregulatory pathways. In the same regard, the findings of the present study were strongly approved by Abo-Aziza et al. [44] that documented a marked decrease in serum TNF-*α* levels at week 2 and week 4, respectively, of transplantation with BM-MSC+albendazole (ABZ) therapy, whereas the level of IL-10 was considerably elevated only at week 4 after transplantation. Additionally, our outcomes are inconsistent with Wei et al. [45] who revealed that BM-MSCs successfully lowered the expression level of TNF-*α* as well as other inflammatory cytokines in blood and hippocampus tissues. Overall, the results of the current study provide evidence for the successful effects of BM-MSCs and IMC in downregulating Th1 cytokine (TNF-*α*), iNOS, MMP-9, and TGF-*β*1 and upregulating Th2 cytokine (IL-10), and all of these effects may have important roles in relieving the manifestations of the experimentally induced rheumatoid arthritis in Wistar rats (Figure 11).

## 5. Summary and Conclusion

Generally, all preceding data proved the validity of BM-MSC+IMC as a promising therapy for RA more than each treatment alone. This was evidenced by their effectiveness in inhibiting paw swelling, reducing anti-CCP concentration, downregulating the proinflammatory Th1 cytokine (TNF-*α*), iNOS, MMP-9, and TGF-*β*1, and upregulating the anti-inflammatory Th2 cytokine (IL-10). Th1 cytokine (TNF-*α*), Th2 cytokine (IL-10), iNOS, MMP-9, and TGF-*β*1 are possible targets of BM-MSCs and IMC to mediate the antiarthritic effects in CFA-induced arthritic rats.

## Figures and Tables

**Table 1:** The forward and reverse primer sequences of various mRNA genes.

Gene	Primer sequence	Amplicon size (bp)
*β*-Actin (housekeeping gene)	F: 5′-TCACCCTGAAGTACCCCATGGAG-3′	151
	R: 5′-TTGGCCTTGGGGTTCAGGGGG-3′	
TNF-*α*	F: 5′-AAAATCCTGCCCTGTCACAC-3′	323
	R: 5′-GCTGAGGTTGGACGGATAAA-3′	
iNOS	F: 5′-ATGGAACAGTATAAGGCAAACACC-3′	220
	R: 5-′GTTTCTGGTCGATGTCATGAGCAAAGG-3′	
MMP-9	F: 5′-CTGGGCTTGATGCCTGTTT-3′	331
	R: 5′-TTGTGGTGGTGCCACTTGA-3′	

## Data Availability

The data used to support the findings of this study are available from the corresponding author upon reasonable request.
